# Bacteriological safety of packaged drinking water sold in Nigeria: public health implications

**DOI:** 10.1186/s40064-015-1447-z

**Published:** 2015-10-26

**Authors:** Olumide A. Odeyemi

**Affiliations:** Ecology and Biodiversity Centre, Institute for Marine and Antarctic Studies (IMAS - Launceston), University of Tasmania, Launceston, Australia; Research and Training Unit, Springforth Scientific Resource Centre, Ikorodu, Lagos Nigeria

**Keywords:** Microbial water safety, Packaged drinking water, Water borne pathogens

## Abstract

**Background:**

Over the past years, there has been increase in packaged water consumption in Nigeria. Although, there are several studies on microbial safety of sachet packaged drinking water, there is no information on prevailing pathogens.

**Findings:**

A comprehensive literature search and meta-analysis of peer reviewed primary studies reported from 2005 for microbiological safety of packaged drinking water sold in Nigeria was conducted using “sachet water”, “bottled water” and “packaged water” and Nigeria as search algorithms in public scientific literature databases. It was observed in this study that *Escherichia* spp., (65.5 %), *Salmonella* spp., (44.8 %), *Bacillus* spp., (44.1 %) and *Staphylococcus* spp. (37.9 %) were more prevailing in the samples.

**Conclusions:**

The high rate of contamination observed is of public health importance. There is need for use of molecular based methods to understand microbial ecology, epidemiology, virulence factors and survival of isolated water borne pathogens in packaged drinking water sold in Nigeria.

## Background

Water is an essential natural resource required by all living organisms. However among these living organisms, human beings tend to use water most for the purposes of drinking, personal, domestic, industrial and recreational uses (Igbeneghu and Lamikanra 2014). Nigeria like other developing nations is faced with problems of potable water supply for its estimated 160 million citizens (Adesiji 2013). As a result of this and other factors, packaged drinking water has been used as alternative drinking water source (Oyedeji et al. 2010). Packaged drinking water is defined as water packaged in cans, plastic sachets and pouches for the main purpose of consumption (Warburton 1993). It is mostly common in low socio economic countries has means of salvaging scarce potable, safe water and to generate income, yet, various studies have shown that some packaged drinking water may not be safe for drinking due to presence of pathogens (Ahmed et al. 2013; Obiri-Danso et al. 2003).

According to Oyedeji et al. (2010), water borne diseases are one of the major public health related problems in developing countries like Nigeria. The ever increasing demand, sale and indiscriminate consumption of packaged drinking water in Nigeria, therefore, poses significant public health risks to the citizens especially individuals with compromised immune systems (Mgbakor et al. 2011). Most producers of packaged drinking water in Nigeria obtain their raw water mostly from sources such as local, municipal piped water or well water and therefore, do not follow specified standards due to lack of the appropriate drinking water technology (Oluyege et al. 2014).

Despite various studies by researchers, there is no information on prevailing pathogens. This study was, therefore, carried out to compile nationally and internationally published articles in order to summarize and compare occurrence of fecal indicator bacteria and potential water borne bacterial pathogens in packaged drinking water in sold in Nigerian cities with a view to identify any available knowledge gap in microbiological safety of packaged drinking water research in Nigeria.

## Methods

A comprehensive literature search and meta-analysis of peer reviewed primary studies reported from 2005 for microbiological safety of packaged drinking water sold in Nigeria was conducted using “sachet water”, “bottled water” and “packaged water” and Nigeria as search algorithms in Web of Science (Science Direct), Google scholar and ProQuest of electronic bibliographic databases. Article selection and inclusion criteria were based on use of standard microbiological methods, detailed description of method of isolation of bacteria, and statement of occurrence of bacterial pathogens. Studies involving fungi, viruses and physico chemical water analysis were excluded.

## Results and discussion

**Fig. 1:**
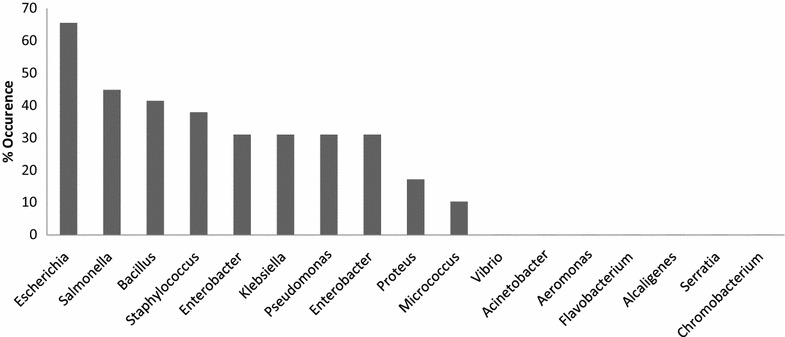
Frequency of occurrence of indicator bacteria and predominate genera in packaged drinking water

This is first study involving systematic review of bacteriological study of packaged drinking water particularly in Nigeria. The study aimed at microbial safety and quality of packaged drinking water sold in Nigeria urban and rural communities. Only 31 published articles met the selection and inclusion criteria for this study. It was observed in this study that *Escherichia* spp., (65.5 %), *Salmonella* spp., (44.8 %), *Bacillus* spp., (44.1 %) and *S. aureus* (37.9 %) were more prevailing in the samples of packaged drinking water analysed by different researchers across the nation (Fig. 1). Some of the predominant genera are pathogenic to human. According to Ahmed et al. (2013), *Aeromonas* spp., *Salmonella* spp., and *Shigella* spp., are potential bacterial pathogens in packaged drinking water. Korzeniewska et al. (2005) stated that bacterial pathogens can survive, multiply to a level that can be detrimental to consumers if stored for long. According to Nigeria Industrial Standard (NIS), the microbiological limit for thermo tolerant coliform or *E. coli* is zero colony forming unit (CFU) in 100 mL of packaged drinking water due to health implications (NIS 2007). This was similar to the report of Venkatesan et al. (2014) who investigated microbiological safety and quality of packaged drinking water in India.

Variations of number of samples analysed was observed while some of the researchers did not state the number of samples analysed. It was observed that the frequency of studies in 2008 and 2012 were greatest and the studies were carried out in 17 cities. Most of the studies were from Imo and Lagos states respectively. It was observed that all the studies used culture based methods for isolation of bacteria which may not give the microbial communities present in the samples.

## Conclusion

The high rate of contamination of packaged drinking water sold in Nigeria is of serious public health concern. There is need for use of molecular based methods to understand microbial ecology, epidemiology, virulence factors and survival of isolated water borne pathogens in packaged drinking water sold in Nigeria. Additionally, National Agency for Food and Drug Administration (NAFDAC), the national regulatory body, should of necessity carry out continuous surveillance and monitoring of registered packaged drinking water.
